# Early-Onset Cancer Incidence Disparities Between Black and White Individuals in the US, 2003-2022

**DOI:** 10.1001/jamanetworkopen.2026.7529

**Published:** 2026-04-16

**Authors:** Wayne R. Lawrence, Andrew P. Loehrer, Eduardo J. Santiago-Rodríguez, Meredith S. Shiels

**Affiliations:** 1 Division of Cancer Epidemiology and Genetics, National Cancer Institute, National Institutes of Health, Rockville, Maryland; 2 Geisel School of Medicine at Dartmouth, Hanover, New Hampshire; 3 Dartmouth Cancer Center, Lebanon, New Hampshire; 4 Department of Surgery, Dartmouth Hitchcock Medical Center, Lebanon, New Hampshire; 5 The Dartmouth Institute for Health Policy and Clinical Practice, Lebanon, New Hampshire; 6 Division of Cancer Control and Population Sciences, National Cancer Institute, National Institutes of Health, Rockville, Maryland

## Abstract

This cross-sectional study examines differences in early-onset cancer rates between Black and White individuals in the US from 2003 to 2022.

## Introduction

Racial differences in cancer incidence are well documented, and cancers attributable to modifiable risk factors—particularly poverty—disproportionately affect Black adults.^1,2^ Early-onset cancers, frequently defined as cancer occurring before age 50 years, have emerged as a major public health concern potentially related to an increased prevalence of metabolic diseases, dietary changes, and earlier cancer screening and detection.^3^ Here, we describe differences in early-onset cancer rates between Black and White individuals in the US from 2003 to 2022.

## Methods

This cross-sectional study used national cancer incidence data from the US Cancer Statistics Database from January 2003 to December 2022. Because our analyses focused on early-onset cancers, the study was restricted to non-Hispanic Black (hereafter, Black) and non-Hispanic White (hereafter, White) individuals aged 20 to 49 years (eMethods in Supplement 1). Race and ethnicity were abstracted from medical records at central cancer registries. The study was deemed non–human participant research and exempt from review and informed consent by the National Institutes of Health’s institutional review board, and followed the STROBE reporting guidelines.

We calculated sex-stratified incidence rates (cases per 100 000) and annual age-adjusted incidence rate ratios (IRRs) comparing Black and White 20- to 49-year-olds for all cancers combined and for leading cancer types using SEER*Stat software version 9.0.41 (National Cancer Institute), age-standardized to the 2000 population.^1^ Joinpoint regression was used to estimate average annual percentage changes (AAPCs) in incidence rates. Two-sided *P* < .05 was considered statistically significant. Data were analyzed from September to November 2025.

## Results

**Figure 1.**
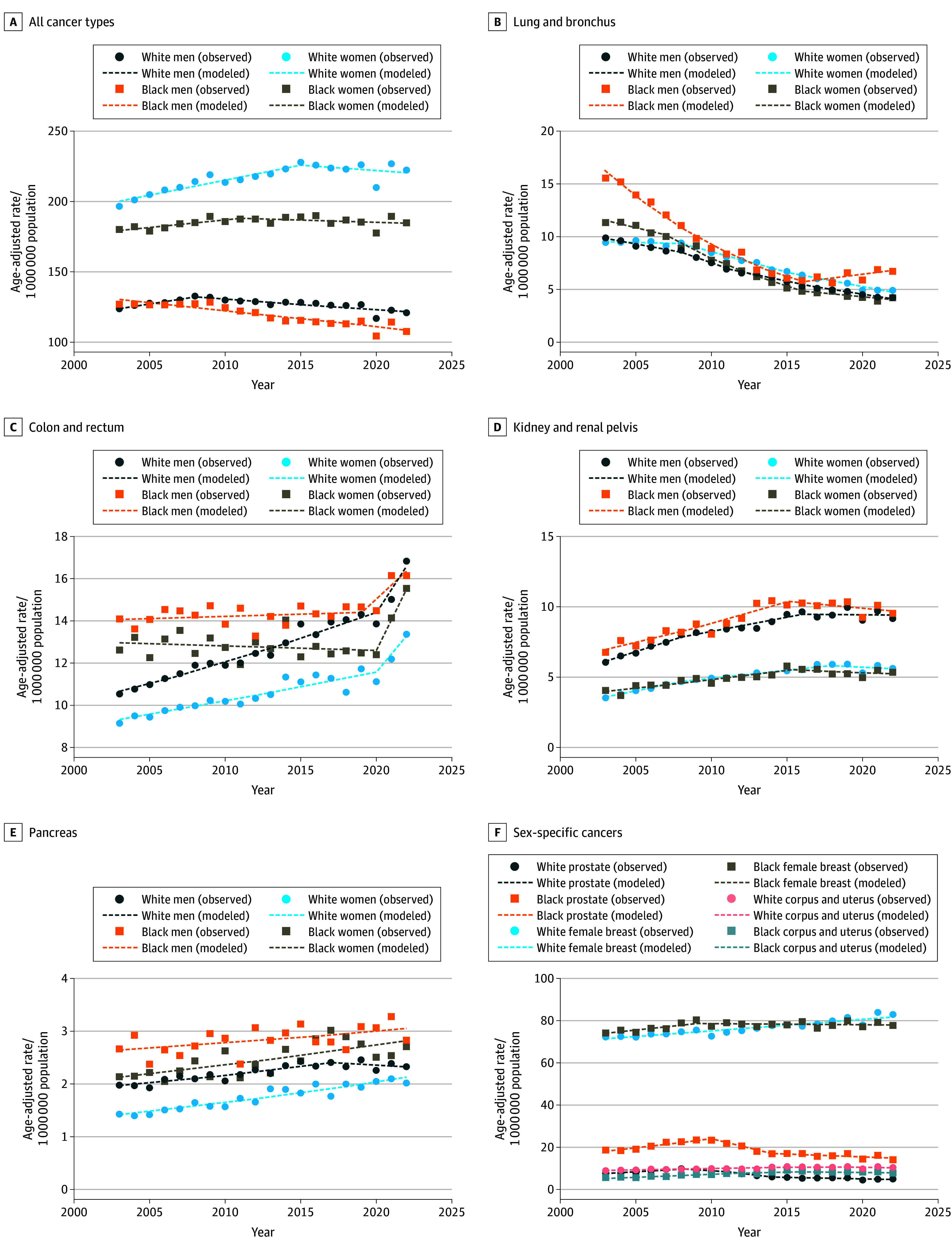
Line Graphs of Trends in Age-Standardized Early-Onset Cancer Incidence Rates for Non-Hispanic Black Individuals and Non-Hispanic White Individuals in the US, 2003 to 2022 Appendix cancers were excluded from colorectal cancer analysis because of classification changes that resulted in rapid, artifactual increases in appendix cancer rates over the study period.

In 2003, the overall early-onset cancer rate was 127.1 cases per 100 000 among Black men and 123.8 cases per 100 000 among White men, decreasing to 107.5 cases per 100 000 (AAPC, −0.7; 95% CI −0.8 to −0.5) and 120.9 cases per 100 000 (AAPC, −0.1; 95% CI, −0.2 to 0.0), respectively, by 2022 (Figure 1). Overall cancer rates among women increased from 2003 (180.1 cases per 100 000 for Black women; 196.6 cases per 100 000 for White women) to 2022 (184.9 cases per 100 000 for Black women; 222.4 cases per 100 000 for White women), although the increase was significant only for White women (AAPC, 0.7; 95% CI, 0.5 to 0.9). The cancer sites with the greatest increasing trends were kidney cancer among Black men (AAPC, 1.8; 95% CI, 1.3 to 2.4) and colorectal cancer among White men (AAPC, 2.3; 95% CI, 2.0 to 2.5). In women, the most rapid increases were uterine cancer among Black women (AAPC, 1.9; 95% CI, 1.4 to 2.3) and kidney cancer among White women (AAPC, 2.5; 95% CI, 2.4 to 3.2).

**Figure 2.**
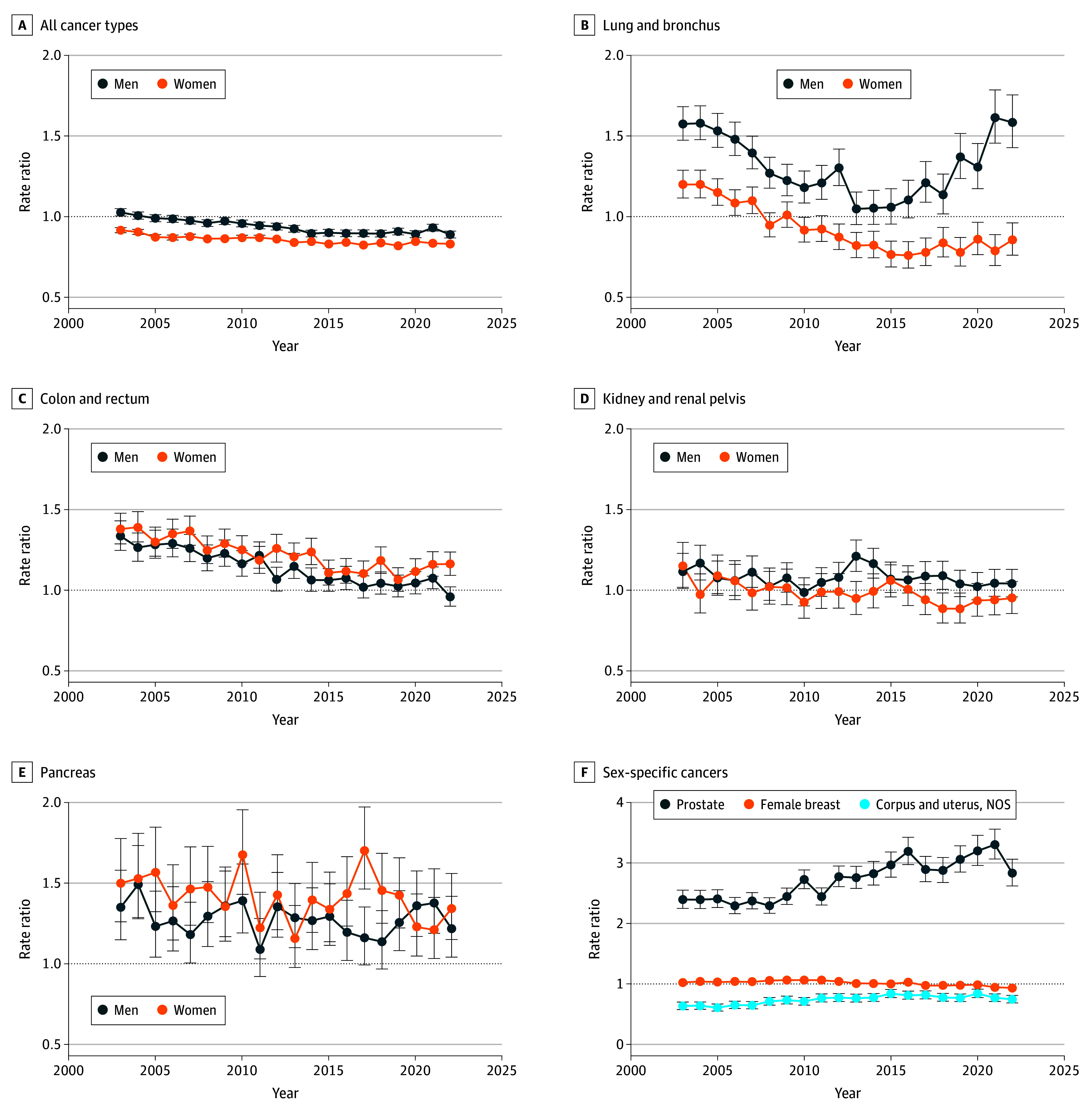
Dot Plots of Early-Onset Cancer Incidence Rate Ratios for Non-Hispanic Black Individuals and Non-Hispanic White Individuals in the US, 2003-2022 Non-Hispanic White individuals are the reference group. Whiskers indicate 95% CIs; in some cases, 95% CIs are too narrow to be visible. Appendix cancers were excluded from colorectal cancer analysis because of classification changes that resulted in rapid, artifactual increases in appendix cancer rates over the study period. NOS indicates not otherwise specified.

The IRRs comparing Black and White men increased for prostate cancer from 2003 (IRR, 2.40; 95% CI, 2.25-2.55) to 2022 (IRR, 2.83; 95% CI, 2.62-3.06) (Figure 2). Breast cancer IRRs decreased from 2003 (IRR, 1.03; 95% CI, 1.00-1.05) to 2022 (IRR, 0.94; 95% CI, 0.91-0.96). Over the period, lung cancer IRRs declined in men from 2003 (IRR, 1.57; 95% CI, 1.47-1.68) to 2013 (IRR, 1.05; 95% CI, 0.95-1.15) and subsequently increased thereafter (2022: IRR, 1.58; 95% CI, 1.43-1.76). Colorectal cancer rates were higher among Black women, although the IRR declined over time from 2003 (IRR, 1.38; 95% CI, 1.29-1.48) to 2022 (IRR, 1.16; 95% CI, 1.09-1.24). From 2003 to 2022, pancreatic cancer rates were higher for Black men (2003: IRR, 1.35; 95% CI, 1.15-1.58; 2022: IRR, 1.22; 95% CI, 1.04-1.42) and Black women (2003: IRR, 1.50 95% CI, 1.26-1.78; 2022: IRR, 1.34; 95% CI, 1.15-1.56). Black women had persistently lower rates of uterine cancer (2003: IRR, 0.64; 95% CI, 0.58-0.70; 2022: IRR, 0.75; 95% CI, 0.69-0.81).

## Discussion

In this cross-sectional study, from 2003 to 2022 among Black and White individuals aged 20 to 49 years, cancer rates declined among men and increased among women. Although early-onset colorectal cancer rates increased in Black and White men and women, potentially in association with increases in screening and detection, racial differences declined.^1,3^ Large increases in colorectal cancer rates in 2021 and 2022 are likely related to new recommendations to start screening at age 45 years.^4^ Recent years have seen an increase in early-onset lung cancer rates among Black men, resulting in rates that were 58% higher in 2022, despite no difference in 2013 to 2016. Cigarette smoking prevalence is comparable between Black and White populations; thus, further research is needed to understand this observed difference.^1^ From 2013 to 2019, there were largely no Black-White difference in breast cancer rates, until 2021 to 2022 when rates were higher in White women, potentially associated with greater screening uptake among the age 40 to 49 years group.^5^ From 2003 to 2022, early-onset pancreatic cancer rates were higher among Black men and women, potentially owing to greater barriers to preventive care for clinical risk factors.^1,2^ Black men have higher prostate cancer rates across the age range.^6^ However, increasing Black-White differences at younger ages may be related to guidelines that recommended prostate cancer screening among Black men younger than 50 years.^6^ In Black and White women, uterine cancer increased from 2003 to 2022, potentially because of increases in obesity and changes in reproductive factors.^1,2^ A limitation is that uterine cancer rates were not adjusted for hysterectomy. Eliminating racial differences in early-onset cancer will require addressing socioenvironmental determinants of risk and increasing equitable access to preventive care.

## Notes

Supplement 1. eMethods eReferences

Supplement 2. Data Sharing Statement
