# Supplementary material for: Early-Onset Cancer Incidence Disparities Between Black and White Individuals in the US, 2003-2022
Source: JAMA Netw Open. 2026 Apr 16;9(4):e267529. doi: 10.1001/jamanetworkopen.2026.7529 (PMC13087818; doi:10.1001/jamanetworkopen.2026.7529)
Supplement: Supplement 1. — eMethods eReferences [file jamanetwopen-e267529-s001.pdf]

Supplemental Online Content

Lawrence WR, Loehrer AP, Santiago-Rodríguez EJ, Shiels MS. Early-onset cancer incidence disparities between Black and White individuals in the US, 2003-2022. *JAMA Netw Open*. 2026;9(4):e267529. doi:10.1001/jamanetworkopen.2026.7529

eMethods

eReferences

This supplemental material has been provided by the authors to give readers additional information about their work.

© 2026 Lawrence WR et al. *JAMA Network Open*.

eMethods

Data Source and Population Characteristics

For this serial cross-sectional study, demographic characteristics and cancer incidence data were ascertained from the US Cancer Statistics Database from January 1, 2003 to December 31, 2022. Data were analyzed from September 2025 to November 2025. Because our analyses focused on early-onset cancers, we restricted the study to non-Hispanic Black (hereafter Black) and non-Hispanic White (hereafter White) adults aged 20-49 years. Race and ethnicity were abstracted from medical records at central cancer registries and categorized based on North American Association of Central Cancer Registries (NAACCR) codes. The present study focused on overall cancer incidence and leading cancer sites (prostate, female breast, lung and bronchus [lung], colon and rectum [colorectal], kidney and renal pelvis [kidney], pancreas, and corpus and uterus [uterine]).[1] Appendiceal cancer was excluded from all colorectal cancer incidence data due to classification changes that resulted in rapid artificial increases over time [2]. This study followed the Strengthening the Reporting of Observational Studies in Epidemiology (STROBE) reporting guidelines.[3] The National Institutes of Health's Institutional review board approval was not needed as data are publicly available.

Statistical Analysis

We calculated sex-stratified incidence rates (per 100,000) and annual age-adjusted incidence rate ratios (IRRs) comparing Black and White 20-49-year-olds for all cancers combined and for leading cancer types using SEER*STAT software (version 9.0.41), age-standardized to the 2000 population. Joinpoint regression was used to estimate average annual percent changes (AAPCs) in incidence rates, excluding 2020 due to artificially low rates during the COVID-19 pandemic.[4]

© 2026 Lawrence WR et al. *JAMA Network Open*.

P values were calculated with the permutation distribution of the test statistic (*P*<0.05 was considered significant, with a 2-sided test).

© 2026 Lawrence WR et al. *JAMA Network Open*.

eReferences

1. Saka AH, Giaquinto AN, McCullough LE, et al. Cancer statistics for African American and Black people, 2025. *CA Cancer J Clin*. Published online February 20, 2025. doi:10.3322/caac.21874

2. Montminy EM, Zhou M, Maniscalco L, et al. Contributions of Adenocarcinoma and Carcinoid Tumors to Early-Onset Colorectal Cancer Incidence Rates in the United States. *Ann Intern Med*. 2021;174(2):157-166. doi:10.7326/M20-0068

3. Ghaferi AA, Schwartz TA, Pawlik TM. STROBE Reporting Guidelines for Observational Studies. *JAMA Surg*. 2021;156(6):577. doi:10.1001/jamasurg.2021.0528

4. Mariotto AB, Feuer EJ, Howlader N, Chen HS, Negoita S, Cronin KA. Interpreting cancer incidence trends: challenges due to the COVID-19 pandemic. *JNCI: Journal of the National Cancer Institute*. 2023;115(9):1109-1111. doi:10.1093/jnci/djad086

© 2026 Lawrence WR et al. *JAMA Network Open*.
