# Supplementary material for: Early-Onset Cancer Incidence Disparities Between Black and White Individuals in the US, 2003-2022
Source: JAMA Netw Open. 2026 Apr 16;9(4):e267529. doi: 10.1001/jamanetworkopen.2026.7529 (PMC13087818; doi:10.1001/jamanetworkopen.2026.7529)
Supplement: Supplement 2. — Data Sharing Statement [file jamanetwopen-e267529-s002.pdf]

Data Sharing Statement

Lawrence. Early-Onset Cancer Incidence Disparities Between Black and White Individuals in the US, 2003-2022. *JAMA Netw Open*. Published April 16, 2026.
doi:10.1001/jamanetworkopen.2026.7529

Data
Data available: No

Additional Information
Explanation for why data not available: De-identified cancer incidence data are available to researchers free of charge in a public use database. To obtain access to the U.S. Cancer Statistics database, you must first obtain SEER data Research Plus access and then submit the U.S. Cancer Statistics Database Request Form.
